# Abdominal endovascular aneurysm repair using an aorto-uni-iliac device resulting in resting lower extremity pain: a case report and discussion on patient selection

**DOI:** 10.1093/jscr/rjae557

**Published:** 2024-08-28

**Authors:** John R Ekblad, Colleen Hupp

**Affiliations:** Department of Surgery, Sky Ridge Medical Center, 10101 RidgeGate Parkway, Lone Tree, CO 80124, United States; Department of Surgery, Sky Ridge Medical Center, 10101 RidgeGate Parkway, Lone Tree, CO 80124, United States

**Keywords:** aorto-uni-iliac stent graft, aorto-uni-iliac, aorto-mono-iliac, AUI, femorofemoral crossover bypass, FCB

## Abstract

Abdominal aortic aneurysms that meet criteria for repair are often managed with endovascular aneurysm repair using a bifurcated two-piece or bifurcated single-body stent. Patients with difficult anatomy, extensive calcifications, complete occlusion of common or external iliac artery, tortuous vessels, or small vessels may require placement of an Aorto-Uni-Iliac (AUI) stent graft. Placement of an AUI stent graft is typically combined with a femorofemoral crossover bypass to ensure adequate perfusion to the contralateral limb. In the elective setting, some literature now supports that select patients with unilateral occlusive common or external iliac disease may be treated with an AUI stent graft alone without femorofemoral crossover bypass. Here, we present a case of a 79-year-old female with an abdominal aortic aneurysms with unilateral occlusive iliac disease managed with an AUI stent graft who subsequently developed rest pain requiring a femorofemoral crossover bypass.

## Introduction

Abdominal aortic aneurysms (AAA) that meet criteria for repair are often managed with endovascular aneurysm repair using a bifurcated two-piece or bifurcated single-body stent. Patients with difficult anatomy, extensive calcifications, complete occlusion of common or external iliac artery, tortuous vessels, or small vessels may require placement of a Aorto-Uni-Iliac (AUI) stent graft [1–4]. Placement of an AUI stent graft is typically combined with a femorofemoral crossover bypass (FCB) to ensure adequate perfusion to the contralateral limb [1, 4, 5]. In the elective setting, some literature now supports that select patients with unilateral occlusive common iliac or external iliac disease may be treated with an AUI stent alone without FCB [2, 3]. This is possible due to the collateral blood supply that develops around the chronic iliac artery occlusion. Major collaterals include the epigastric vessels, pelvic collaterals, circumflex iliac, and lumbar collaterals [3].

## Case report

**Figure 1:**
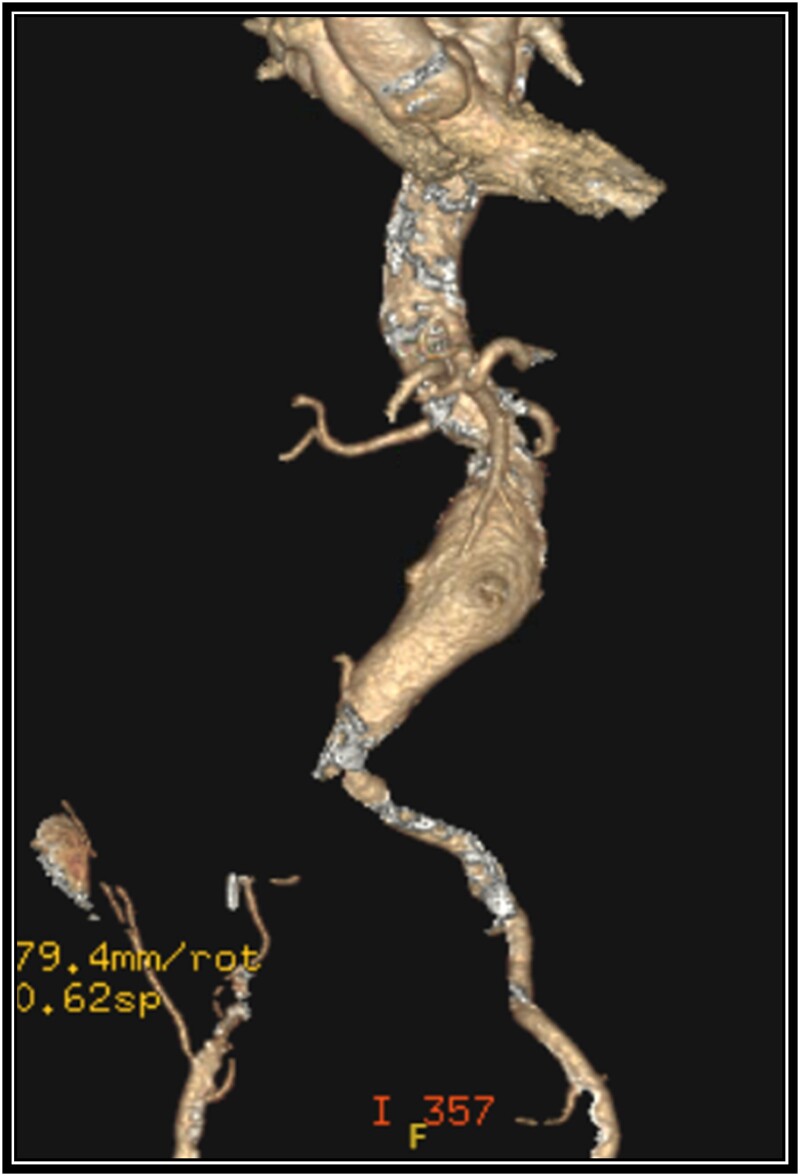
Preoperative 3-D reconstruction based on CTA imaging demonstrating an infrarenal abdominal aortic aneurysm and complete occlusion of the right common iliac artery with distal reconstitution.

A 79-year-old female with past medical history of peripheral arterial disease, chronic obstructive pulmonary disease on 4 L oxygen baseline, history of tobacco use, and hypertension presented with an enlarging abdominal aortic aneurysm measuring 5.2 cm and chronic occlusion of the right common iliac artery secondary to vascular disease. The abdominal aneurysm was 3.5 cm 6 years ago, but a recent MRI for back pain found it had grown to 5.2 cm. The patient lives a sedentary lifestyle, primarily uses a wheelchair, ambulates a limited amount with a walker, and experiences dyspnea on exertion. She denies claudication symptoms. A CTA was completed for preoperative planning (Fig. 1).

**Figure 2:**
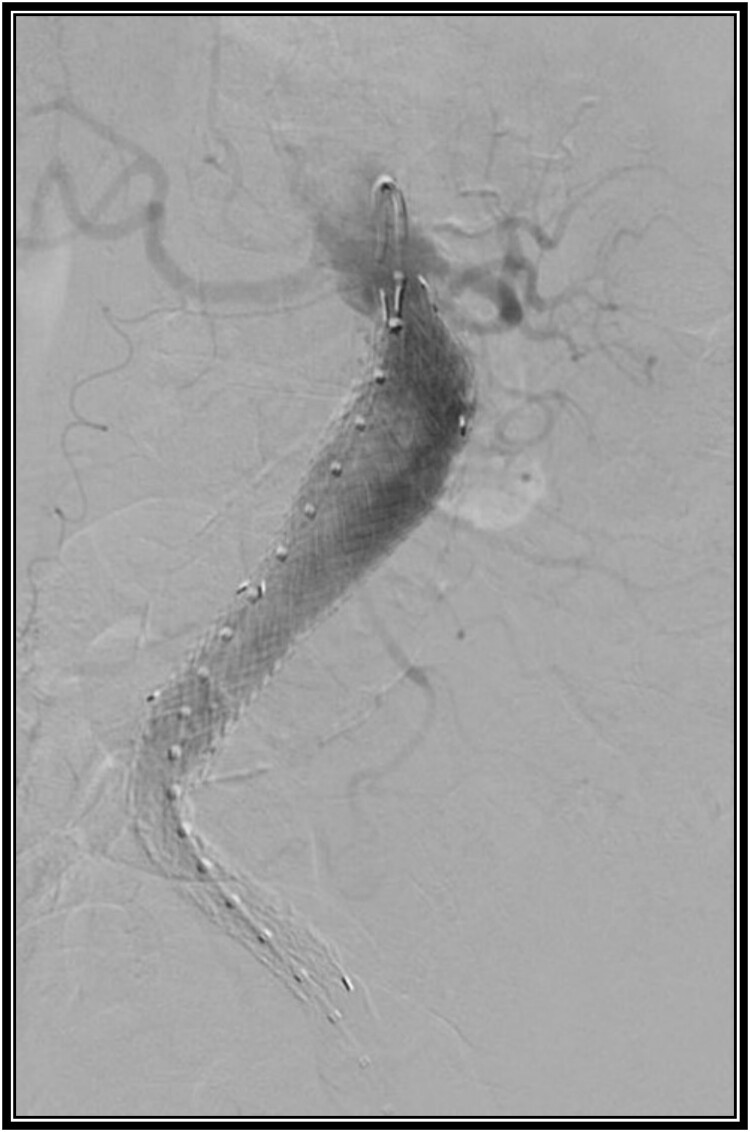
Intraoperative angiogram after placement of an aorto-uni-iliac stent graft.

Given the patient has extensive comorbidities, it was decided to repair her AAA via an endovascular approach. Since she had complete occlusion of the right common iliac artery and lives a sedentary lifestyle without claudication symptoms, it was decided to perform a left AUI stent graft repair without FCB. This procedure also included a left common femoral endarterectomy given her significant atherosclerotic disease. Due to stenosis of her left common iliac artery, an 8 mm × 60 mm balloon was used to dilate this area to allow passage of the sheath. A 27 mm × 16 mm × 10 cm limb was modified to be used as the exclusion device. The device was deployed below the renal arteries. A 16 mm × 12 mm × 12 cm extension limb was placed, terminating above the left internal iliac artery. A type 1a endoleak was seen. A 28 mm × 3.3 cm aortic cuff was deployed just below the renal arteries. This resolved the type 1a endoleak. There was a small type 2 endoleak noted on the final angiogram (Fig. 2). The patient had a good recovery and was discharged on postoperative Day 1.

CTA on postoperative Day 24 showed the AUI stent was patent with no endoleaks. Reconstitution of the right common femoral artery can be seen at the junction with the external iliac artery. Upon clinic follow-up on postoperative Day 27, the patient complained of new rest pain and claudication in the right lower extremity. The patient was subsequently taken for an elective right common femoral endarterectomy and left to right FCB using a 6 mm ringed PTFE graft. The addition of this bypass resolved the patient’s right lower extremity rest pain.

## Discussion

In the case presented, the patient appeared to be a good candidate for an AUI stent without FCB to repair her AAA given her right common iliac artery was chronically occluded, she did not have any claudication symptoms, and she lived a sedentary lifestyle. The patient’s primary collateral circulation likely originated from the lumbar arteries, which were covered with the AUI stent graft while her remaining collaterals were not developed enough to maintain adequate perfusion resulting in claudication symptoms.

There are multiple advantages to performing AUI stent graft without FCB when repairing an AAA. There is less operative time, less blood loss, reduced exposure to anesthesia, and no additional risk of steal syndrome [3]. Performing an FCB adds an additional incision to the contralateral thigh with placement of an additional graft. This adds to the risk of wound complications and introduces another graft that could become infected or thrombose [3].

A study by Elkassaby *et al.* [3] presented a select group of six patients with chronic uni iliac occlusion managed with an AUI stent without FCB. This group was compared to a group of 34 patients who were managed with AUI stent with FCB. Patients who were selected for AUI stent without FCB had absent rest pain, no tissue loss, no claudication, and ABI > 0.5 in the chronically occluded limb. All patients managed with AUI stent without FCB tolerated the procedure well; only two patients developed mild claudication symptoms. They concluded that select patients could benefit from the less invasive AUI without FCB.

Rogers *et al.* [2] demonstrated in a retrospective review that 7 of 15 patients with AAA and unilateral iliac artery occlusion were managed successfully with an AUI stent without a FCB. These patients had no ischemic symptoms prior to repair. These patients did not develop claudication symptoms of either lower extremity at follow-up, which was believed to be in part due to their sedentary lifestyle and well-developed collaterals.

Despite the advantages of the AUI without FCB, there is a risk of covering a portion of the collateral circulation to the contralateral leg. If a large enough portion of this collateral circulation is compromised, this can result in ischemic symptoms of the contralateral leg. As highlighted previously, when selecting patients for AUI without FCB for AAA, it is important to take into consideration comorbidities, lifestyle, ischemic/claudication symptoms, chronicity of iliac occlusion, and ABIs.

## Conclusion

This case highlights that determining who will require an FCB at the index operation when utilizing an AUI stent graft for AAA repair in patients with unilateral occlusive iliac disease remains a challenge, and multiple factors need to be taken into consideration when making this decision. If an AUI stent graft without FCB is chosen, we recommend close clinical follow-up to determine if any ischemic symptoms are present and to determine if further intervention is warranted.
